# Drought tolerance during reproductive development is important for increasing wheat yield potential under climate change in Europe

**DOI:** 10.1093/jxb/ery226

**Published:** 2018-06-12

**Authors:** Nimai Senapati, Pierre Stratonovitch, Matthew J Paul, Mikhail A Semenov

**Affiliations:** Rothamsted Research, West Common, Harpenden, UK

**Keywords:** Climate change, drought stress, drought tolerance, ideotype optimization, reproductive development, wheat yield potential, yield stability

## Abstract

Drought stress during reproductive development could drastically reduce wheat grain number and yield, but quantitative evaluation of such an effect is unknown under climate change. The objectives of this study were to evaluate potential yield benefits of drought tolerance during reproductive development for wheat ideotypes under climate change in Europe, and to identify potential cultivar parameters for improvement. We used the Sirius wheat model to optimize drought-tolerant (DT) and drought-sensitive (DS) wheat ideotypes under a future 2050 climate scenario at 13 contrasting sites, representing major wheat growing regions in Europe. Averaged over the sites, DT ideotypes achieved 13.4% greater yield compared with DS, with higher yield stability. However, the performances of the ideotypes were site dependent. Mean yield of DT was 28–37% greater compared with DS in southern Europe. In contrast, no yield difference (≤1%) between ideotypes was found in north-western Europe. An intermediate yield benefit of 10–23% was found due to drought tolerance in central and eastern Europe. We conclude that tolerance to drought stress during reproductive development is important for high yield potentials and greater yield stability of wheat under climate change in Europe.

## Teaser

Modelling predicts that drought tolerance during reproductive development is important for higher yield potentials (up to 37%) and greater yield stability for wheat under future climate change in Europe.

## Introduction

Wheat (*Triticum aestivum* L.) is one of the key staple crops for global food security, providing about 20% of the total dietary calories and protein needs, with about 730 million tons of annual production from around 2.1 million km^2^ harvested area globally (Shiferaw *et al.*, 2013; FAO, 2016). In Europe, wheat is the most widely grown food crop, contributing 34% to global wheat production from about 27% of the global wheat area (FAOSTAT, 2014). The ongoing climate changes, characterized by increase in frequency and severity of climatic extreme events and adverse weather conditions, threaten global wheat production including Europe (Asseng *et al.*, 2015; Stratonovitch and Semenov, 2015; Zampieri *et al.*, 2017). Among different adverse weather conditions and climatic extreme events, drought is one of the major abiotic stresses that limit crop production (Lipiec *et al.*, 2013; Basu *et al.*, 2016; Fahad *et al.*, 2017). The frequency and intensity of drought stresses are predicted to increase under future climate change in Europe, particularly in central and southern Europe (Dai, 2013; Kovats *et al.*, 2014). Thus, the risk of yield losses and even crop failure will increase in Europe under the future climatic conditions (Trnka *et al.*, 2014, 2015).

Drought affects both source and sink strengths, leading to source- and sink-limited yield reduction of up to 92% in wheat, depending on the crop growth stage, duration, and intensity of drought stress (Farooq *et al.*, 2014; Semenov *et al.*, 2014). The drought stress, particularly during reproductive development, reduces grain number in wheat (Dolferus *et al.*, 2011; Dong *et al.*, 2017; Ma *et al.*, 2017). Reproductive development includes development of floral and reproductive structures, formation of the male and female gametophytes, fertilization, and primary grain setting. The potential grain number is set first by the number of flower initials that are formed on the spike. Potential grain number could be reduced by premature abortion of florets due to drought stress (Dolferus *et al.*, 2011, 2013). The potential grain number in wheat can be reduced considerably further even by a short spell of drought during meiosis and gametogenesis due to male and female sterility (Lalonde *et al.*, 1997; Ji *et al.*, 2010; Dolferus *et al.*, 2011; Barber *et al.*, 2015; Onyemaobi *et al.*, 2017). The young microspore stage is the most vulnerable to drought stress in wheat, leading to reproductive sterility (Ji *et al.*, 2010; Dolferus *et al.*, 2013). Malfunction and irreversible abortion of male and female reproductive organs and gametophytes are the main reasons for drought-induced male and female infertility in wheat (Saini, 1997; Ji *et al.*, 2010; de Storme and Geelen, 2014; Dong *et al.*, 2017; Onyemaobi *et al.*, 2017), and reduced viability of gametophytes due to drought stress decreases the final fertile grain number (Lalonde *et al.*, 1997; Saini, 1997; Ma *et al.*, 2017).

Developing wheat cultivars tolerant to drought stress during reproductive development is currently a big challenge for wheat breeders (Cattivelli *et al.*, 2008; Mwadzingeni *et al.*, 2016). Drought tolerance is not a qualitative trait, but a complex quantitative plant trait, which is controlled by numerous genes and other plant traits, with minor individual contributions (Blum, 2010; Dolferus *et al.*, 2013; Hu and Xiong, 2014; Serba and Yadav, 2016). Breeding of drought-tolerant wheat cultivars suffers from complex multi-trait and polygenic control of drought tolerance, high genotype and environment (G×E) interactions, low heritability, and difficulty in mass screening of plant traits and genes (Cattivelli *et al.*, 2008; Fleury *et al.*, 2010; Hu and Xiong, 2014). Experimental analysis of potential yield benefits of drought tolerance during reproductive development under future climate change and identification of the target traits for improvement are difficult due to the complexity in designing such experiments in the real world for climates that can only be predicted with a high degree of uncertainty.

Donald (1968) proposed an alternative breeding approach, ‘breeding of crop ideotypes’, in which breeders select plant ideotypes based on knowledge of crop physiology for improvement of plant traits under the target environment and then breed for them, rather than breeding for ‘defect elimination’ and ‘selection for yield’. A crop ideotype is a virtual idealized crop, or a crop model, which is expected to produce a greater quality and quantity of grain when developed as a cultivar. Process-based ecophysiological crop models are the most powerful tools that help to (i) deconvolute a complex trait, such as drought tolerance, into a list of simpler component traits suitable for further analyses and linking with phenotype and genotype, and then breeding; (ii) evaluate the performance of different plant traits under future climatic conditions and thus assist in discovering and prioritizing target traits for improvement; (iii) search optimal combinations and trade-offs between target traits; (iv) design ideotypes optimized for target environments and guide plant breeders towards the most relevant targets (Hammer *et al.*, 2005; Semenov and Halford, 2009; Reynolds *et al.*, 2011; Sylvester-Bradley *et al.*, 2012; Martre *et al.*, 2015; Rötter *et al.*, 2015; Gouache *et al.*, 2017). In our study, we used Sirius, which is a process-based wheat model coupled with an ideotype optimization framework. Sirius was calibrated and validated for different modern wheat varieties, and performed well under diverse climatic conditions across Europe, USA, Australia and New Zealand, including free-air CO_2_ enrichment experiments (Jamieson *et al.*, 1998, 2000; Lawless and Semenov, 2005; Martre *et al.*, 2006; Stratonovitch and Semenov, 2010; He *et al.*, 2012; Semenov *et al.*, 2014; Asseng *et al.*, 2015). Although a few studies have projected the effects of common water limitation and drought stresses on wheat yield potentials under future climate change in Europe, the quantitative effects of reproductive stage drought stress on grain number and subsequently on grain yield have not been considered before (Semenov and Stratonovitch, 2010; Semenov and Shewry, 2011; Stratonovitch and Semenov, 2015; Trnka *et al.*, 2014, 2015). A few experimental studies have reported the performance of reproductive stage drought-tolerant and drought-sensitive wheat germplasms under current climate, but quantitative potential yield benefits from drought tolerance during reproductive development under future climatic conditions are mostly unknown (Ji *et al.*, 2010; Dong *et al.*, 2017; Ma *et al.*, 2017). In the present study, the important mechanism for the effect of drought stress on grain number during reproductive development was incorporated into Sirius for evaluation of yield benefits of drought tolerance during reproductive development under future climate change in Europe.

The main objectives of the present modelling study were (i) to assess potential yield benefits of drought tolerance during reproductive development for wheat ideotypes under future climate change in Europe, and (ii) to identify cultivar parameters that could be related to wheat traits to achieve high yield potential under climatic change.

## Materials and methods

### Target site and future 2050 climate

**Fig. 1.**
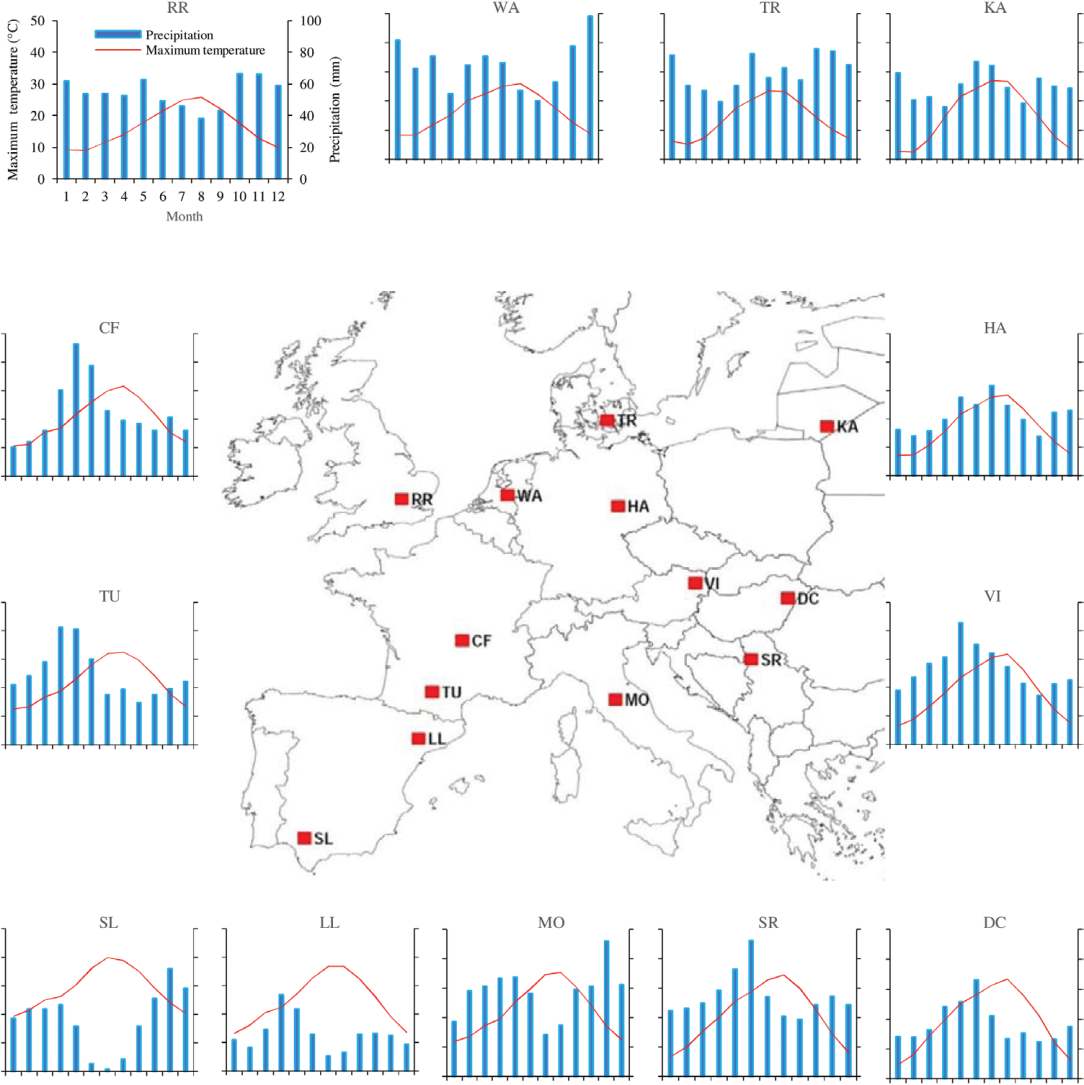
Locations of 13 selected study sites, representing major wheat growing regions across Europe. Mean maximum temperature and mean monthly precipitation are shown for the future 2050 climate scenario (based on HadGEM2 and RCP8.5). (This figure is available in colour at *JXB* online.)

For the present study, 13 sites across Europe were selected, representing the major and contrasting wheat growing regions in Europe, from Spain in the south to Denmark in the north, and Hungary in the east to the UK in the west (Fig. 1). Table 1 shows the detailed site characteristics, typical locally cultivated wheat varieties, and the sowing dates. Out of six wheat cultivars, Cartaya and Creso are spring wheat, whereas the others are winter wheat cultivars. The future climate in 2050 was based on a global climate model (GCM), HadGEM2, from the CMIP5 ensemble (Taylor *et al.*, 2012) for the Representative Concentration Pathway 8.5 (RCP8.5). The RCP8.5 combines assumptions about high population and modest technological improvements, leading to high energy demand with the highest greenhouse gas (GHG) concentration and a radiative forcing of +8.5 W m^−2^ (Riahi *et al.*, 2011). Climate projections from HadGEM2 were downscaled to the local daily weather by using the LARS-WG 6.0 weather generator (Semenov and Stratonovitch, 2010, 2015). The atmospheric CO_2_ concentration in 2050 was increased to 541 ppm following the RCP8.5 scenario. For each site, the climatic scenario contains 100 years of site-specific daily weather, which was used as input for optimization and evaluation of ideotype performances (Fig. 1; Table 1).

**Table 1.** Site characteristics of the selected wheat growing regions across Europe

No.	ID	Site	Country	Latitude (°)	Longitude (°)	Average air temperature (°C)^*a*^	Precipitation (mm year^−1^)^*a*^	Cultivar^*b*^	Sowing date^*b*^
1	SL	Seville	Spain	37.42	−5.88	22.1	434	Cartaya	30 December
2	LL	Lleida	Spain	41.63	0.60	18.0	311	Creso	25 November
3	MO	Montagnano	Italy	43.30	11.80	16.0	686	Creso	25 November
4	TU	Toulouse	France	43.62	1.38	16.7	595	Thesee	20 November
5	SR	Sremska	Serbia	45.00	19.51	15.2	649	Thesee	15 November
6	CF	Clermont-Ferrand	France	45.80	3.10	14.7	536	Thesee	15 November
7	DC	Debrecen	Hungary	47.60	21.60	14.2	441	Thesee	18 October
8	VI	Vienna	Austria	48.23	16.35	14.5	643	Thesee	20 October
9	HA	Halle	Germany	51.51	11.95	12.7	509	Claire	20 October
10	RR	Rothamsted	UK	51.80	−0.35	12.2	653	Mercia	10 October
11	WA	Wageningen	Netherlands	51.97	5.67	12.3	779	Claire	20 October
12	KA	Kaunas	Lithuania	54.88	23.83	10.5	605	Avalon	25 October
13	TR	Tylstrup	Denmark	57.20	9.90	10.6	721	Avalon	18 October

^*a*^ Future 2050 climate scenario (based on HadGEM2 and RCP8.5).

^*b*^ Typical local cultivated wheat varieties and the sowing dates.

### Sirius model

Sirius is a process-based wheat model with an optimization framework based on evolutionary algorithms with self-adaptation. This framework allows designing ideotypes and optimization of cultivar parameters for target environments. A detailed description of the Sirius model can be found elsewhere (Jamieson *et al.*, 1998, 2000; Jamieson and Semenov, 2000; Brooks *et al.*, 2001; Lawless *et al.*, 2005; Martre *et al.*, 2006; Semenov and Halford, 2009; Semenov *et al.*, 2009, 2014; Stratonovitch and Semenov, 2010). In brief, Sirius consists of submodels that describe soils, water and nitrogen (N) uptake, photosynthesis, biomass accumulation and partitioning (leaf, stem, grain, and root), phenological development, including responses to limitation of N supply, along with adverse climatic effects such as heat and drought stress.

### Photosynthesis and biomass accumulation

Photosynthesis and biomass production are calculated on a daily basis as the product of intercepted photosynthetically active radiation (PAR) and radiation use efficiency (RUE), limited by temperature and water stress. Radiation interception is related to leaf area index (LAI) via the Lambert–Beer law, with a default extinction coefficient of 0.45. In Sirius, RUE is proportional to atmospheric CO_2_ concentration, with an increase of 30% for a doubling in CO_2_ concentration for a C_3_ crop (e.g. wheat) (Vanuytrecht *et al.*, 2012). The shortage of N limits leaf area, and hence light interception and biomass production.

### Canopy development

Canopy development is described as a series of leaf layers associated with individual mainstem leaves. Leaf area development in each layer is simulated by a thermal time submodel, and actual leaf area is calculated using a simple limitation rule. Phenological development is calculated from the mainstem leaf appearance rate and final leaf numbers, with the latter determined by responses to daylength and vernalization. Maximum area of flag leaf (*A*) influences the rate of canopy expansion and the maximum achievable LAI. The duration of leaf senescence is expressed in thermal time and linked to the rank of the leaf in the canopy. Total canopy senescence synchronizes with the end of grain filling. Leaf senescence could be accelerated by shortage of N to sustain green leaves and grain filling, or by abiotic stress, viz. temperature or water stress. One of the strategies to increase grain yield is to extend the duration of leaf senescence and maintain green leaf area longer after anthesis, termed ‘stay green’ (*SG*).

### Phenology and grain development

Phyllochron (*Ph*), daylength response (*Pp*) and duration of grain filling (*Gf*) are directly related to phenological development of wheat. *Ph* is the thermal time required for the appearance of successive leaves, and is a major driver of phenological development. *Ph* and *Pp* together determine the rate of crop development and the date of flowering and maturity. *Gf* is defined as a cultivar-specific amount of thermal time that needs to be accumulated to complete grain filling. During grain filling, assimilates for the grain are available from two sources viz. (i) new biomass produced from intercepted radiation after anthesis, and (ii) water-soluble or labile carbohydrates stored mostly in the stem before anthesis.

### Root growth and soil water uptake

Soil is described as a cascade of 5-cm layers up to a user-defined depth. Roots continue to grow until reaching a soil-dependent maximum depth or until anthesis, whichever occurs first. Each soil layer contains root-available (water potential <−1.5 MPa) and -unavailable (water potential >−1.5 MPa) water, depending on its water retention characteristics. Only a proportion of available soil water can be extracted by plants from each layer of the root zone on any day depending on efficiency of water extraction (λ) and rate of root water uptake (*Ru*).

### Impact of water limitation on biomass production and grain yield

Water limitation adversely affects both source (carbohydrate production) and sink strength (grain filling) in the plant. Photosynthesis and biomass production are reduced by water limitation. New biomass production decreases proportionally to the response of photosynthesis to water stress (*Wsa*), defined as *Wsa*=SF^β^, where SF is a stress factor and β is a cultivar-independent constant. The rate of leaf senescence increases under water limitation by a factor, maximum acceleration of leaf senescence (*Wss*), that modifies daily increment of thermal time. Earlier leaf senescence will reduce grain yield by reducing grain size due not only to reduction in intercepted radiation and photosynthesis, but also to reduction in translocation of the labile plant reserve carbohydrate to the grain due to premature termination of grain filling driven by early leaf senescence.

### Impact of drought stress on grain number during reproductive development

A simple mechanism was implemented in the current version of Sirius to account for the effect of drought stress on grain number during reproductive development by using a drought stress factor (DSF). The DSF was calculated as a ratio of actual transpiration (*T*_a_) to potential transpiration (*T*_p_) during reproductive development. However, linking different reproductive development stages (floral formation and development, meiosis and gametogenesis, fertilization, etc.) to corresponding plant growth stages for modelling is difficult as they often happen within a short period and vary depending on the climatic conditions and abiotic stresses. For example, meiosis in wheat often coincides with booting stage, but meiosis within a single floret can last only for 1–2 d, whereas meiosis within an ear and plant could be extended by 3–5 d (Bennett *et al.*, 1972; Saini and Aspinall, 1982; Barber *et al.*, 2015; Onyemaobi *et al.*, 2017). In general, around 10 d before the flowering date until 5 d after the flowering date is the most critical period for reproductive development including fertilization and vulnerability to drought stress in wheat (Dolferus *et al.*, 2011; Wise *et al.*, 2011; Barber *et al.*, 2015). In the present study, the impact of drought stress on reproductive development was implemented in Sirius for an average of 15 d, viz. 10 d before the flowering date and 5 d after the flowering date.

In the absence of drought stress, the sink capacity of the grains (*Y*_pot_, g m^−2^) is set as the product of the potential number of grains and the potential weight of an individual grain:

$$Y_{\text{pot}}={\text{DM}}_{\text{ear}}\times N_{\text{pot}}\times W_{\text{pot}}$$

where DM_ear_ (g m^−2^) is the dry matter accumulated in ears prior to anthesis, *N*_pot_ (grains g^−1^) is the maximum number of grains per unit of ear dry mass, and *W*_pot_ (g grain^−1^) is the potential weight of a single grain. In the absence of abiotic stress, the default parameter values of *N*_pot_=100 grains g^−1^ and *W*_pot_=50 mg are large enough to provide sufficient sink capacity to accommodate newly produced and translocated biomass. Therefore, in the absence of drought stress, grain yield will be determined by the source capacity of the crop.

To account for the effect of drought stress during reproductive development, the number of fertile grains produced per unit of ear dry matter is reduced when DSF falls below a threshold, DSGNT. The reduction factor of grain number (*R*, dimensionless) is calculated as:

$$R={\text{DSGNR}}_{\text{Max}},\text{ if DSF}\leq \text{DSGNS}$$

$$R={\text{DSGN}}_{\text{Max}}+S\times (\text{DSF}-\text{DSGNS}),\text{ if DSGNS}<\text{DSF}<\text{DSGNT}$$

R=1, if DSF>DSGNT

where DSGNR_Max_ is maximum drought stress grain number reduction, DSGNS is drought stress grain number reduction saturation, DSGNT is drought stress grain number reduction threshold, and *S* is the slope of the grain number reduction, and *S*=(1−DSGNR_Max_)/(DSGNT−DSGNS). The value of parameters for drought-sensitive cultivars were selected as DSGNT=0.9, DSGNS=0.3 and DSGNR_Max_=0.2. The actual number *N* (grains g^−1^) of grains per unit of ear dry matter is the product of the potential number of grains and the drought reduction factor:

$$N=N_{\text{pot}}\times R$$

### Target traits for improvement under future climate change

Plants deploy many strategies and adaptations for survival and complete the life cycle in different ways under water stress, viz. drought escape, avoidance, and tolerance (Farooq *et al.*, 2014; Yadav and Sharma, 2016). Drought tolerance is a complex trait controlled by many individual plant traits with small contributions (Semenov and Halford, 2009; Fleury *et al.*, 2010; Hu and Xiong, 2014). A total of eight cultivar parameters related to drought escape, avoidance, and tolerance traits were selected for improvement to maximize yield potential of future wheat cultivars under targeted climatic conditions (Table 2). By adjusting *Ph* and *Pp*, the rate of crop development could be increased, which could shorten the duration of the vegetative growth phase and help to escape terminal drought by early flowering and maturity. Early flowering is an important trait for drought escape while maintaining potential yield (Shavrukov *et al.*, 2017). *Gf* has been suggested as a possible trait for increasing grain yield in wheat (Evans and Fischer, 1999). However, increasing *Gf* could be in conflict with yield improvement due to water stress under terminal drought. The rate of canopy expansion and the maximum LAI could be adjusted by altering the cultivar parameter *A*. This in turn will change the pattern and quantity of light interception and transpiration, and therefore, will affect crop growth, water use efficiency, and finally grain yield. A decrease in *A* could help to avoid drought stress by reducing transpiration and root water uptake. *Ru* is an important root trait affecting temporal patterns and total amount of water uptake in water-limited environments (Manschadi *et al.*, 2006). A faster root water uptake reduces current water stress experienced by the plant, but could be risky for successful completion of the life cycle under terminal drought. On the other hand, slower water uptake with a likely drought at the end of the growing season is less risky for drought avoidance and may achieve on average higher yields. *SG* is a drought tolerance trait that enables plants to retain more green leaves longer after anthesis and improve potential yield under drought stress (Silva *et al.*, 2001; Triboi and Triboi-Blondel, 2002; Luche *et al.*, 2015; Christopher *et al.*, 2016). *Wsa* and *Wss* are two additional drought tolerance traits that could help in increasing yield potential under drought stress (Semenov and Halford, 2009; Semenov *et al.*, 2014).

**Table 2.** Sirius cultivar parameters used for designing wheat ideotypes under the future 2050 climate scenario (based on HAdGEM2 and RCP8.5), and genetical variation observed in those parameters

No.	Parameters	Symbol	Unit	Range used in model optimization	Genetical variation observed for wheat	Reference
1	Phyllochron	*Ph*	°C day	80–130	≤20%	Ishag *et al.* (1998), Mosaad *et al.* (1995)
2	Day length response	*Pp*	Leaf h^−1^ day length	0.05–0.70	9.74–107.40a	Kosner and Zurkova (1996)
3	Duration of grain filling	*Gf*	°C day	500–900	≤40%	Akkaya *et al.* (2006), Charmet *et al.* (2005), Robert *et al.* (2001)
4	Maximum area of flag leaf	*A*	m^2^ leaf m^−2^ soil	0.003–0.01	≤40%	Fischer *et al.* (1998), Shearman *et al.* (2005)
5	Stay green	*SG*	—	0.0–1.5		
6	Rate of root water uptake	*Ru*	%	1.0–7.0	Large variation	Asseng *et al.* (1998), Manschadi *et al.* (2006)
7	Response of photosynthesis to water stress	*Wsa*	—	0.1–2.1		
8	Maximum acceleration of leaf senescence due to water stress	*Wss*	—	1.2–1.9		

^*a*^ Varietal difference in number of days till heading under long and short day conditions varied between 9.74 and 107.40 in a photoperiodic response experiment.

### Designing wheat ideotypes under future climate change

In the present study, a crop ideotype was defined as a set of cultivar parameters that would deliver optimal yield performance in a target environment. The ideotype will produce maximum possible yield when developed as a cultivar. A wheat ideotype was characterized by eight cultivar parameters, controlling crop growth, development, and responses to drought stresses, which are summarized in Table 2 and described in the section ‘Target traits for improvement under future climate change’. We designed a drought-sensitive (DS) and a drought-tolerant (DT) ideotype for each site separately (Tables 2 and 3). The DS ideotype is sensitive to drought stress during reproductive development, in which grain number could be reduced depending on the severity of drought stress. In contrast, a DT ideotype is tolerant, or insensitive to any such drought stress during reproductive development. A total of eight cultivar parameters were optimized during the ideotype design, where initial cultivar parameters were the same for both DS and DT ideotypes at a given site (Tables 2, 3).

**Table 3.** Optimized parameter values of drought-tolerant (DT) and drought-sensitive (DS) wheat ideotypes under the future 2050 climate scenario (based on HadGEM2 and RCP8.5) at 13 sites across major wheat growing regions in Europe

Ideotype	Optimized parameter							
	*Ph*	*Pp*	*Gf*	*A*	*SG*	*Ru*	*Wsa*	*Wss*
	(°C day)	(leaf h^−1^ day length)	(°C day)	(m^2^ leaf m^−2^ soil)		(%)		
SL (Seville, Spain)								
Initial^*a*^	105.0	0.2000	550.0	0.0065	0.5000	3.00	0.50	1.27
* *DS final	97.4	0.1152	827.1	0.0069	0.1244	7.00	0.10	1.28
* *DT final	129.9	0.1247	900.0	0.0100	1.3461	3.07	0.10	1.20
LL (Lleida, Spain)								
Initial	90.0	0.6000	650.0	0.0030	0.5000	3.00	0.50	1.27
* *DS final	102.9	0.1046	761.9	0.0044	0.8404	7.00	0.10	1.20
* *DT final	127.6	0.1157	900.0	0.0100	1.2294	2.15	0.10	1.20
MO (Montagnano, Italy)								
Initial	90.0	0.6000	650.0	0.0030	0.5000	3.00	0.50	1.27
* *DS final	109.3	0.1165	839.0	0.0089	1.0216	6.33	0.10	1.20
* *DT final	129.7	0.1378	900.0	0.0100	1.3384	3.97	0.10	1.20
TU (Toulouse, France)								
Initial	94.0	0.4000	650.0	0.0040	0.5000	3.00	0.50	1.27
* *DS final	129.2	0.0500	900.0	0.0100	1.0971	7.00	0.10	1.20
* *DT final	130.0	0.0500	900.0	0.0100	0.9760	6.79	0.10	1.20
SR (Sremska, Serbia)								
Initial	94.0	0.4000	650.0	0.0040	0.5000	3.00	0.50	1.27
* *DS final	120.5	0.0500	899.4	0.0049	1.3993	7.00	0.10	1.26
* *DT final	129.7	0.0500	900.0	0.0100	1.3865	5.88	0.10	1.20
CF (Clermont-Ferrand, France)								
Initial	94.0	0.4000	650.0	0.0040	0.5000	3.00	0.50	1.27
* *DS final	115.9	0.0500	829.4	0.0100	0.9802	7.00	0.10	1.20
* *DT final	129.8	0.0500	900.0	0.0100	1.1370	6.08	0.10	1.20
DC (Debrecen, Hungary)								
Initial	94.0	0.4000	650.0	0.0040	0.5000	3.00	0.50	1.27
* *DS final	115.1	0.0500	803.2	0.0060	1.3279	7.00	0.10	1.20
* *DT final	129.9	0.0500	900.0	0.0100	0.9615	5.12	0.10	1.20
VI (Vienna, Austria)								
Initial	94.0	0.4000	650.0	0.0040	0.5000	3.00	0.50	1.27
* *DS final	129.7	0.0500	900.0	0.0100	1.0178	7.00	0.10	1.20
* *DT final	130.0	0.0500	900.0	0.0100	1.5000	5.94	0.10	1.20
HA (Halle, Germany)								
Initial	110.0	0.5000	650.0	0.0070	0.5000	3.00	0.50	1.27
* *DS final	110.3	0.0500	859.8	0.0044	0.8355	7.00	0.10	1.20
* *DT final	130.0	0.0503	899.5	0.0100	1.0085	4.16	0.10	1.20
RR (Rothamsted, UK)								
Initial	107.0	0.5300	650.0	0.0075	0.5000	3.00	0.50	1.27
* *DS final	130.0	0.0500	899.9	0.0100	0.8288	7.00	0.10	1.20
* *DT final	130.0	0.0890	900.0	0.0100	0.9612	5.91	0.10	1.20
WA (Wageningen, Netherlands)								
Initial	110.0	0.5000	650.0	0.0070	0.5000	3.00	0.50	1.27
* *DS final	129.9	0.0500	900.0	0.0100	0.8039	7.00	0.10	1.20
* *DT final	129.9	0.0500	900.0	0.0099	0.9955	6.81	0.10	1.20
KA (Kaunas, Lithuania)								
Initial	90.0	0.6500	650.0	0.0065	0.5000	3.00	0.50	1.27
* *DS final	130.0	0.0968	715.9	0.0061	1.4786	6.37	0.10	1.20
* *DT final	130.0	0.0595	900.0	0.0100	1.3428	4.89	0.10	1.20
TR (Tylstrup, Denmark)								
Initial	90.0	0.6500	650.0	0.0065	0.5000	3.00	0.50	1.27
* *DS final	126.1	0.0678	792.6	0.0039	1.4974	5.32	0.10	1.39
* *DT final	129.9	0.0874	900.0	0.0100	1.3877	3.09	0.10	1.20

^*a*^ Initial values represent the parameter values of the current wheat varieties in a given site.

*A*, maximum area of flag leaf; *Gf*, duration of grain filling; *Ph*, phyllochron; *Pp*, day length response; *Ru*, rate of root water uptake; *SG*, stay green; *Wsa*, response of photosynthesis to water stress; *Wss*, maximum acceleration of leaf senescence.

### Ideotype optimization

Both ideotypes, viz. DS and DT, were optimized to achieve high yield potentials in target environments. An evolutionary search algorithm with self-adaptation (EASA) was used in Sirius to optimize cultivar parameters in a high-dimensional parameter space with a complex fitness function for the best performance of crop ideotypes (Schwefel and Rudolph, 1995; Stratonovitch and Semenov, 2010). The EASA is considered as a universal search optimization method. The main advantage of EASA compared with genetic algorithms is that it does not require tuning control parameters during the search, where predefined heuristic rules are unavailable or difficult to formulate (Beyer, 1995; Bäck, 1998; Semenov and Terkel, 2003). The EASA optimized cultivar parameters by randomly perturbing (mutating) their values and testing their performance in the target environment. At each step of optimization, 16 new candidate ideotypes were generated from a ‘parent’ by perturbing its cultivar parameters randomly within the predefined parameters’ limits (Table 2). The parameter ranges were based on calibration of Sirius for modern cultivars allowing for variations reported in the literature for existing wheat germplasms (Semenov and Halford, 2009; He *et al.*, 2012; Semenov and Stratonovitch, 2013; Semenov *et al.*, 2014). Yield was calculated for each of 16 candidates for 100 years under future climatic conditions. Ideotypes with the coefficient of variation (CV) of yield exceeding 10% were excluded from the selection process and the candidate with the highest mean yield over 100 years was selected as a ‘parent’ for the next step of optimization. The stopping rules of optimization process were: (i) no further improvement in yield potential is possible, and (ii) parameter convergence and parameter optimal state are achieved.

In an additional set of simulations, we relaxed the criteria of yield variance during the optimization process, excluding ideotypes with CV exceeding 15% from the selection process. The purpose of increasing CV was to evaluate the effect of increased yield variance during optimization on the performance of ideotypes.

### Simulation set-up

We used Sirius version 2015, which is available from https://sites.google.com/view/sirius-wheat/. A single soil-water profile (*Hafren*) with a total available water capacity of 177 mm was used at all sites to eliminate site-specific soil effects from the analysis. The soil profile was filled with the maximum available water capacity at sowing. An increase of 10% in RUE was assumed for wheat by 2050 (Zhu *et al.*, 2010). Both the wheat ideotypes (DS and DT) were optimized for the maximum possible yield under future climatic conditions (2050) for the selected sites. All simulations were assumed to be water-limited, but no N limitation was considered. Additionally, the effects of an adverse weather event of heat stress around flowering on grain number and size (Stratonovitch and Semenov, 2015) were excluded in the present study to simulate only the effect of drought stress under future climate change.

## Results

### Simulated yield potential of wheat ideotypes under target future 2050 climate

**Fig. 2.**
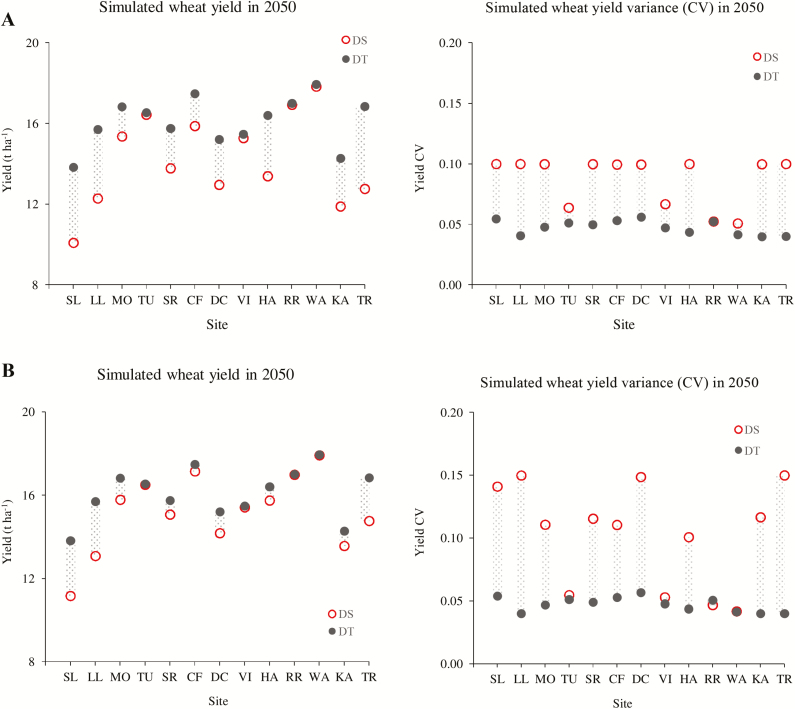
Wheat yield and yield coefficient of variance (CV) of drought-sensitive (DS) and drought-tolerant (DT) ideotypes optimized under the future 2050 climate scenario (based on HadGEM2 and RCP8.5) at 13 sites, representing major wheat growing regions in Europe. The yield CV during model optimization was limited to 10% (A) and 15% (B). (This figure is available in colour at *JXB* online.)

The upper two panels in Fig. 2 show simulated wheat yield and yield variance (coefficient of variation; CV) for DT and DS ideotypes under the future climate change in 2050 at 13 sites across major wheat growing regions in Europe, when yield CV was limited to 10% during model optimization. Averaged over the sites, the DT ideotype achieved 13% higher mean yield (16.1 t ha^−1^) compared with DS (14.2 t ha^−1^). Averaged over both ideotypes, the highest wheat yield was found at WA (17.9 t ha^−1^), followed by RR, CF, and TU, whereas the lowest yield was observed at SL (12.0 t ha^−1^), followed by KA. All the other sites had a yield in the range of 14–16 t ha^−1^. However, there were strong site effects on ideotype performances at individual sites.

When the site-specific performance of individual wheat ideotypes was compared, the highest yields (17–18 t ha^−1^) were achieved for the DT ideotype at WA, RR, TR, CF, TU, and MO, whereas lowest yields (~14 t ha^−1^) were obtained at SL and KA. Intermediate yields of 15–16 t ha^−1^ were found at the rest of the sites. On the other hand, maximum yields (16–18 t ha^−1^) for DS were obtained at WA, RR, TU, and CF, whereas minimum yield was found at SL (~10 t ha^−1^), followed by KA and LL (~12 t ha^−1^). A medium wheat yield of around 13–15 t ha^−1^ was observed at other sites. When the site-specific performance of DT was compared with DS, simulated wheat yields of DT were 28–37% greater compared with DS at SL, LL, and TR, with the highest potential yield benefit of 37% for DT at SL. In contrast, a similar magnitude of wheat yield was obtained for both DT and DS ideotypes in the range 15–18 t ha^−1^ at WA, RR, VI, and TU, where the difference in yield potentials between two ideotypes was ≤1%. The mean wheat yield of DT was 10–23% greater compared with DS at the other six sites.

### Stability of simulated wheat yield potential under future climate

Averaged over sites, mean yield variance (CV) over 100 years’ simulations was 0.05 (CV=0.04–0.06) and 0.09 (CV=0.05–0.10) for DT and DS ideotypes, respectively (Fig. 2). The result indicates 44% smaller yield variance for DT compared with the DS ideotype. Among individual sites, four out of 13 sites had almost equal yield variance (CV~0.05) for both the ideotypes, viz. TU, VI, RR, and WA. In the rest of the sites, CV of wheat yield potential for DT was 50% smaller compared with DS.

### Effect of limiting yield variance during optimization process on simulated yield potentials and variability

The lower two panels in Fig. 2 show simulated wheat yields and yield CV for DT and DS ideotypes across 13 European sites when yield CV over 100 years was limited to 15% during model optimization. When yield CV was increased from 10% to 15% during the optimization process, no effect was found on the results (yields and CV) for the DT ideotype across different sites as reported above. Averaged over the sites, the minimum and mean wheat yields of DS increased by 11 and 7%, respectively, whereas maximum yield did not increase either at individual sites or averaged over different sites. At the same time, the maximum and mean CV of wheat yield for the DS ideotype increased by 50 and 18%, respectively.

### Optimization of wheat ideotype parameters under target future 2050 climate

Averaged over the sites and ideotypes, optimized values for *Ph*, *Gf*, *A*, and *SG* were found to be increased from their initial values by 29, 36, 87, and 122%, in which optimized values were 10, 8, 54, and 87% greater, respectively for the DT ideotype compared with DS (Table 3). In contrast, the optimized value of *Ru* increased by 95% compared with its initial value, but optimized values were 54% greater for the DS ideotype compared with DT. An equally high optimized *Ru* value (~7%) was obtained at almost all the sites for the DS ideotype. Optimized values of the rest of the three parameters, viz*. Pp*, *Wsa* and *Wss*, decreased by 83, 80, and 4%, respectively, where no difference (≤2%) was found between ideotypes, except for *Pp*. Similar to the simulated wheat yields, strong site effects were found on the optimized parameter values and between ideotypes in an individual site.

The highest differences in the optimized parameter values between ideotypes were found for *Ph* (33%), *Pp* (63%), *Gf*, (26%), *A* (156%), *SG* (982%), and *Ru* (226%) at SL, LL, KA, and TR (Table 3). For all these parameters, optimized values were greater for DT compared with DS, except for *Pp* and *Ru*, where both the values were greater for DS compared with DT. An overall minimum or almost no difference in these parameter values between ideotypes was found at WA, RR, VI, and TU. An intermediate ideotype effect was obtained for the same parameters at the rest of the sites, with greater improved values for DT compared with DS. There were no ideotype and site effects for the other two parameters, viz. *Wsa* and *Wss*, where almost constant model optimization effects were obtained (80% for *Wsa* and ~4% for *Wss*).

## Discussion

Overall, higher yield potential of the DT compared with the DS ideotype under future climatic condition could be linked to a greater number of grains for the DT ideotype. The number of fertile grains setting during reproductive development was reduced by drought stress for the DS ideotype, depending on the level of drought stress at different sites, whereas the primary grain setting number remained unaffected for DT due to drought tolerance during reproductive development. Reduction in grain number decreased the total sink capacity resulting in reduced yield potentials for DS. Adverse effects of drought stress on the primary grain setting number during reproductive development have been reported by different experimental studies for common wheat germplasms/lines/cultivars (Lalonde *et al.*, 1997; Ji *et al.*, 2010; Dong *et al.*, 2017; Ma *et al.*, 2017; Onyemaobi *et al.*, 2017) and reviewed for different cereals including wheat (Saini, 1997; Dolferus *et al.*, 2011; de Storme and Geelen, 2014). These studies also found some wheat germplasms tolerant to drought stress during reproductive development, indicating the possibility of improvement in drought tolerance through genetical adaptation. Our study shows that drought tolerance during reproductive development is an important trait for achieving high wheat yield potential under future climate change in Europe.

Another reason for higher yield potential of the DT ideotype compared with DS could be overall better optimized cultivar parameters contributing to a greater source and sink capacity under drought stress, and a direct and indirect contribution to drought tolerance. For example, *SG*, *Gf*, and *A* were greater in the DT ideotype compared with DS. A high *SG* value implies delaying leaf senescence and greater plant capacity to maintain more active photosynthetic tissues longer under water stress during anthesis and grain filling, and is thus considered one of the most important traits for drought tolerance (Cattivelli *et al.*, 2008; Farooq *et al.*, 2014; Luche *et al.*, 2015; Christopher *et al.*, 2016). Greater number of fertile grains per ear, increased average grain weight, and high total yield were reported for different crop cultivars, including wheat, with the stay green trait or genotypes (Silva *et al.*, 2003; Foulkes *et al.*, 2007; Luche *et al.*, 2013). The use of the stay green character in future wheat breeding programmes would result in significant genetic progress for tolerance to terminal drought stress and high yield (Luche *et al.*, 2015). An extended grain filling period (a high *Gf* value) increases grain yield by increasing light interception for more photosynthesis and production of more carbohydrate to be translocated directly to the developing grains. It also increases the possibility of completion of re-translocation of labile carbohydrate reserves to the grains (Semenov and Halford, 2009; Semenov and Stratonovitch, 2013). Duration of the grain filling period is an important trait to improve through breeding for increasing wheat yield potentials under future climate change (Evans and Fischer, 1999; Semenov *et al.*, 2014). Greater *A* increases the potential LAI, which in turn increases light interception, photosynthesis, carbohydrate production, and finally grain yield (Semenov and Stratonovitch, 2013). Increasing both leaf area and grain filling period could increase yield potential further under future climatic conditions for drought-tolerant wheat cultivars. However, greater leaf area and longer grain filling period could conflict with drought avoidance for drought-sensitive cultivars. Our results of relatively smaller increase in optimized values of *A* and *Gf* for DS show that increasing *A* and *Gf* is a trade-off between avoiding drought by reducing transpiration and achieving higher yield potential for the DS ideotype. *Ph* was smaller in the DS ideotype compared with DT, indicating a relatively higher chance for DS to escape any terminal drought by early flowering and maturity due to the shorter vegetative stage. Early flowering and maturity are important drought escape traits for many crops including wheat (Yadav and Sharma, 2016; Shavrukov *et al.*, 2017). Greater *Ru* values for DS compared with DT indicate that drought-sensitive cultivars could take up soil water faster to avoid current drought stress. But, faster water uptake could be risky for DS to complete the life cycle successfully under severe drought at the end of the growing season. On the other hand, drought-tolerant cultivars would take up soil water slower in favour of successful completion of the life cycle with a likely terminal drought. Drought stress generally reduces crop yield through decreased photosynthesis and increased leaf senescence (Yadav and Sharma, 2016; Fahad *et al.*, 2017). Decreased response of photosynthesis to water stress (*Wsa*) and reduced maximum acceleration of leaf senescence (*Wss*) are important traits for drought tolerance (Semenov and Halford, 2009; Semenov and Stratonovitch, 2013). We found reduced (4–80%) optimized values for both *Wsa* and *Wss*, but the respective values were almost equal across sites and ideotypes. Our result indicates that both the cultivar parameters/traits are important for high yield potentials under future climatic conditions, irrespective of tolerance or sensitivity to reproductive stage drought stress. A high genetic variation in *Ph* (Mosaad *et al.*, 1995; Ishag *et al.*, 1998), *Pp* (Kosner and Zurkova, 1996), and *Ru* (Asseng *et al.*, 1998; Manschadi *et al.*, 2006) was found in wheat, whereas up to 40% variation in genotypes was observed in *Gf* (Robert *et al.*, 2001; Charmet *et al.*, 2005; Akkaya *et al.*, 2006) and *A* (Fischer *et al.*, 1998; Shearman *et al.*, 2005) for wheat, indicating the possibility of future improvements through wheat breeding.

The overall high yield potentials of DT and DS ideotypes and their differences under future climatic conditions could be considered as the interactive effects between drought tolerance/sensitivity during reproductive development and eight cultivar parameters optimized for high yield potential under the target climatic condition. Our results demonstrate the importance of the drought tolerance trait during reproductive development in achieving higher yield potentials for the DT ideotype, along with an extra beneficial effect of improved cultivar parameters, linked with high yield and drought tolerance directly and indirectly, whereas results for the DS ideotype, where selected cultivar parameters were not able to be optimized to the same extent as for DT, show that drought sensitivity during reproductive development limits optimization of those parameters. However, optimized cultivar parameters in the DS ideotype compensated for the potential yield loss due to drought sensitivity during reproductive development at least to some extent. Thus, the present study reveals that improved cultivar parameters, such as decreased leaf senescence, reduced response of photosynthesis to water stress and increased root water uptake, may increase yield potentials also for drought-sensitive cultivars. Different studies reported similar importance of drought tolerance during reproductive development and different improved plant traits for high yield potentials of different crops including wheat (Manschadi *et al.*, 2006; Cattivelli *et al.*, 2008; Ji *et al.*, 2010; Farooq *et al.*, 2014; Semenov *et al.*, 2014; Yadav and Sharma, 2016; Shavrukov *et al.*, 2017).

The highest yield benefits (28–37%) due to drought tolerance were obtained mainly in southern Europe (e.g. SL and LL). This result could be explained by the high probability of drought stress during reproductive development under future climate change, and the highest differences in the optimized parameter values (e.g. *SG*, *Gf*, *A*, *Ph*, *Pp*, and *Ru*) between the ideotypes at those sites. Similarly, a medium yield benefit of 10–23% for DT in most of the central and eastern European sites (e.g. MO, SR, DC, and HA) could be due to a most likely medium drought stress during reproductive development under future climatic conditions and an intermediate difference in optimized parameter values in favour of the DT ideotype. In contrast, a minimum or no yield benefit for DT compared with DS was found only at a few sites in north-western and central-western Europe (e.g. RR, WA, and TU), characterized with a low probability of, or no, severe drought stress during reproductive development under future climate change. In these sites, almost equal optimized cultivar values were obtained for both the DT and DS ideotypes. This is in accordance with the minimum or no difference in wheat yield potentials due to ideotypes at those sites. It is worthy of note that we found the highest yield benefits (28–37%) due to drought tolerance at one additional site, TR (Denmark), but it was characterized by a good annual precipitation (721 mm year^−1^) (Figs 1, 2; Table 1). We also found no yield benefit due to drought tolerance at one extra site, VI (Austria), but it was characterized by a medium annual precipitation (643 mm year^−1^) (Figs 1, 2; Table 1). Our results demonstrate that the adverse impact of drought stress on yield potential during reproductive development could happen locally at some sites even with good annual precipitation, for example in Denmark. Drought stress during reproductive development is a dynamic, but a short-term, event (~10–15 d), which depends on many abiotic and biotic factors at that time. Thus, a drought-tolerant wheat cultivar would be important for high yield potential under climate change even in northern Europe (e.g. Denmark). The results for the DS ideotype on decreased wheat yield potentials under future climate change in most of the European sites are in line with Trnka *et al.* (2014, 2015), who predicted that there would be less adaptation options for wheat under future climate change in Europe, with a high risk of loss of yield due to increased adverse extreme events including drought.

Another important finding of the present study was that drought-tolerant wheat ideotypes would have greater yield stability under future climatic conditions, whereas yield stability of drought-sensitive ideotypes would be substantially lower. These results were further supported by the fact that the yield potentials of the DT ideotype were not affected across different sites even when the selection criteria of yield variance (CV) was increased to 15% during the optimization process. On the other hand, mean yield potential of the DS ideotype increased (7%) at the cost of reducing yield stability (18%) further. This indicates that there would be a trade-off between yield potential and yield stability for drought-sensitive wheat cultivars.

We did not account for a detailed mechanism for the adverse effects of drought stress on grain number during reproductive development for wheat at a molecular level; for example, change in hormonal composition and gene expression (Ji *et al.*, 2010, 2011; de Storme and Geelen, 2014; Farooq *et al.*, 2014; Dong *et al.*, 2017; Onyemaobi *et al.*, 2017). However, introduction of a relatively simple description in Sirius for a reduction in grain number due to drought stress during reproductive development was found to be effective for quantitative evaluation of the potential yield benefits from drought tolerance in wheat under future climatic conditions. We conclude that drought tolerance during reproductive development is important for high yield potentials with high yield stability of wheat under future climate change in most of the wheat growing regions in Europe, with predicted maximum potential yield benefits of 28–37% in southern Europe and 10–23% in central and eastern Europe. Introduction of drought tolerance during reproductive development, along with improvements in cultivar parameters linked with increased yield and drought tolerance directly or indirectly, is the key to achieving higher wheat yield potentials along with higher yield stability under future climatic conditions in Europe. Our results identified the important cultivar parameters and their optimal combination for future improvement to achieve high yield potentials under climate change in Europe, viz. grain filling duration, phyllochron, leaf area, stay green, rate of root water uptake, photosynthetic response to water stress, and maximum acceleration of leaf senescence due to water stress.
